# Salvage of a Near-Total Penile Amputation following Urinary Fistulization and Carbapenemase-Producing *Klebsiella pneumoniae* Infection with a Composite ALT Flap and Vascularized Fascia Lata

**DOI:** 10.1055/s-0041-1735649

**Published:** 2021-09-14

**Authors:** Ricardo Horta, Margarida Mendes, Diogo Barreiro, Alexandre Almeida, Mariana Jarnalo, Sérgio Teixeira, Rui Pinto

**Affiliations:** 1 Department of Plastic and Reconstructive Surgery and Burn Unity, Centro Hospitalar Universitário São João, Faculty of Medicine, University of Porto, Porto, Portugal; 2 Department of Urology, Centro Hospitalar Universitário São João, Faculty of Medicine, University of Porto, Porto, Portugal

**Keywords:** penile amputation, salvage, KPC infection, ALT flap, vascularized fascia lata

## Abstract

Reconstruction of complex penile defects is always challenging, as some defects are not possible to reconstruct with skin or mucosa grafts, and even local flaps may be precluded in complex wounds. We present a case of a 63-year-old otherwise healthy man, who underwent transurethral resection of the prostate for benign prostatic hyperplasia. After the procedure, he developed panurethral necrosis with consequent stricture. Three urethroplasties for reconstruction of the bulbar and distal urethra using buccal mucosa grafts, a preputial flap, and penile skin were performed by urology team in different institutions, but serious urinary fistulization and carbapenemase-producing *Klebsiella pneumoniae* (KPC) infection translated in a chronic wound, urethra necrosis, and near-total penile amputation. A composite anterolateral thigh flap and vascularized fascia lata were used with success together with a perineal urethroplasty in different stages, improving the ischemic wound condition. The extended segment of fascia lata was used for Buck's fascia replacement and circumferential reinforcement to cover the erectile bodies of the penis. The postoperative period was uneventful and after 12 months, there were no signs of recurrence or wound dehiscence. He was able and easily adapted to void in a seated position through the perineal urethrostomy that was made. To the best of our knowledge, this procedure has not been reported previously as a salvage procedure in a fistulizated and KPC infected penis, but it may be considered to avoid penile amputation in chronic infected and intractable wounds.

Reconstruction of complex penile defects including urethra, cavernous bodies, and penile/scrotal skin defects is quite challenging. Management of these defects usually comprises surgical reconstruction to restore phallus function and aesthetics. Some defects are not possible to reconstruct with skin or mucosa grafts, and even local flaps may be precluded for previous surgery, trauma, or complex wounds. 1 Good quality of life and functional outcomes have been reported with distant flaps such as the radial forearm free flap, but it has an unfavorable donor-site scar location and is contraindicated in the absence of collateral circulation of the hand. A good and emerging alternative is the anterolateral thigh (ALT) pedicled flap. This flap has the advantages of a better donor-site location and of being pedicled, avoiding the need for microsurgical anastomosis. 2

In some complex infected wounds, it may be useful as a salvage procedure to avoid penile amputation, as the case presented here.

## Case Report

**Fig. 1:**
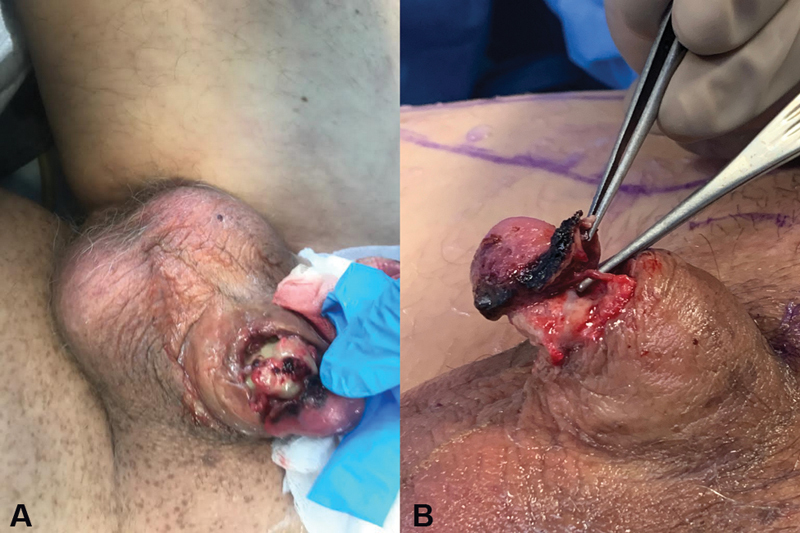
( **A** ) Chronic wound, urethra necrosis, and near-total penile amputation caused by urinary fistulization and carbapenemase-producing *Klebsiella pneumoniae* infection. ( **B** ) Near-total amputation of the glans.

**Fig. 2:**
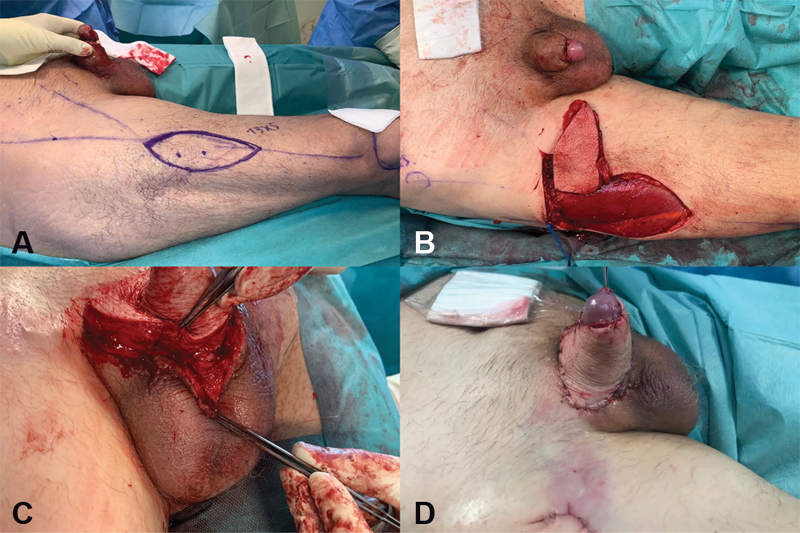
( **A** ) A composite anterolateral thigh flap and vascularized fascia lata was used to improve the ischemic wound condition. ( **B** ) The flap was tunnelized under the rectus femoris and a groin subcutaneous tunnel. ( **C** ) The extended segment of fascia lata was used for Buck's fascia replacement and circumferential reinforcement to cover the erectile bodies of the penis. ( **D** ) Immediate postoperative result.

A 63-year-old otherwise healthy man underwent transurethral resection of the prostate in 2014 for benign prostatic hyperplasia. After the procedure, he developed panurethral necrosis with consequent stricture. Two urethroplasties for reconstruction of the bulbar and distal urethra using buccal mucosa grafts were performed in 2016 and 2017 by urology team in another hospital. Two months after the last reconstructive procedure, the stricture relapsed and a dilation was performed, resulting in a satisfactory urinary flow for 6 months. He underwent another urethroplasty using a preputial flap in a different institution and kept urinary catheterization for additional 6 months. After catheter removal, several fistulas in the penile urethra were found. The patient was under various antibiotic regimens and underwent cystostomy. He presented to our emergency service with penile infection, dehiscence of the balanopreputial sulcus, and partial necrosis of the ventral and lateral aspects of the glans, cavernous bodies, and distal penis ( Fig. 1 ). Empiric antibiotherapy was administered and later adjusted to the microbiological and antibiotic susceptibility testing results. A magnetic resonance imaging confirmed necrosis of the left aspect of the glans, inflammation of the subcutaneous tissues, and possibly a urinoma. A biopsy ruled out squamous cell carcinoma. On December 9, 2019, he underwent surgical debridement of the unviable tissues and reconstruction using a right ALT flap based on two musculocutaneous perforators, extended laterally to the vastus lateralis muscle to include a segment of fascia lata and elevated as a composite flap ( Fig. 2 ). The main pedicle-descending branch of the lateral circumflex femoral artery was dissected until its origin and isolated to preserve the motor nerve to the vastus lateralis muscle and rectus femoris muscle. The entire length of the perforator flap pedicle was 16 cm. The flap was tunnelized under the rectus femoris and a groin subcutaneous tunnel.

The extended segment of fascia lata was used for Buck's fascia replacement, as a layer of deep fascia for circumferential reinforcement and to cover the erectile bodies of the penis. The glans was kept in place given its satisfactory perfusion by the end of the procedure, despite near-total amputation.

**Fig. 3:**
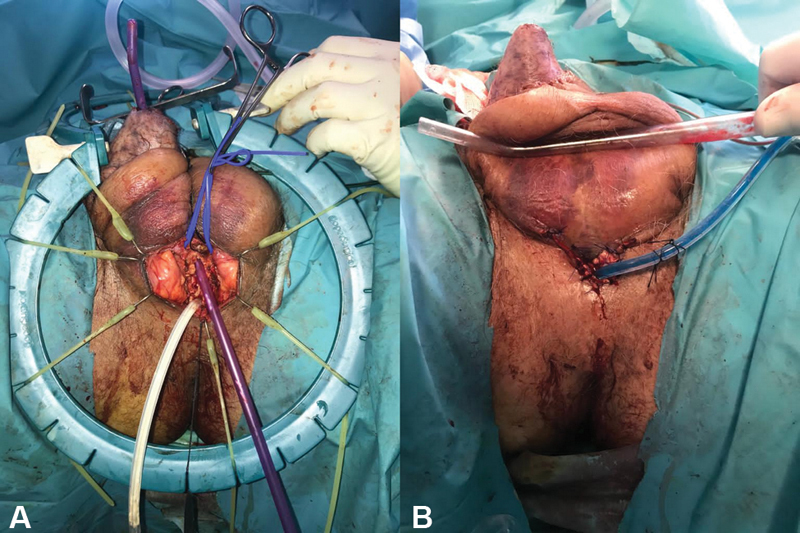
( **A** ) Full urethra necrosis was found. ( **B** ) A perineal urethrostomy was performed by urology team to create a permanent opening into the urethra through an incision in the skin of the perineum.

**Fig. 4:**
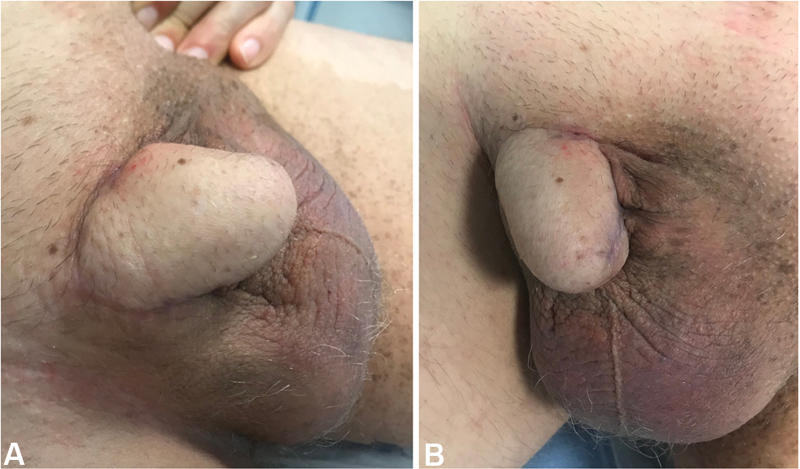
Result after 12 months. ( **A** ) Lateral view and ( **B** ) frontal view.

In the postoperative period, the flap showed good perfusion, however, the glans presented with venous congestion and developed necrosis during the following days. After extensive multidisciplinary discussion, the urethra was considered irreparable to allow voiding through the end of the penis. One week later, he was reoperated for surgical debridement of necrotic tissues of the glans, and a perineal urethrostomy was performed by the urology team to create a permanent opening into the urethra through an incision in the skin of the perineum ( Fig. 3 ). After the second procedure, the patient had purulent drainage by the scrotum basis: a *Klebsiella pneumoniae* sensible to ertapenem, meropenem, and amikacin was isolated and the patient was treated accordingly, with good response to the treatment. Finally, on January 20, 2020, the patient underwent a revisional procedure for flap remodeling. He had a good postoperatory evolution, the urinary diversion (suprapubic cystostomy) was clamped, and the urethral catheter was taken out 3 weeks postreconstruction. After the new opening for urine to pass was created in the perineum, he was able and easily adapted to void in a seated position, maintaining urinary continence. He was discharged on February 14, 2020. After 12 months, there were no signs of recurrence or wound dehiscence. Erectile function was not present until last observation, and a urology appointment was scheduled for planning penile prosthesis insertion. Fat defatting/thinning was unnecessary due to the initial extensive and deep defect. The patient was satisfied with the flap bulk and donor-site scar. A good aesthetic result was seen ( Fig. 4 ).

## Discussion

One-stage reconstruction of complex penile defects presents reconstructive challenges to the surgeon. The selection of proper technique depends on the size and the status of the wound, the condition of local tissues, and donor-site morbidity. When grafts and local flaps are inadequate or unavailable, other options should be considered including microsurgical flap transfers, the appendix, and intestinal segments. 1 It also depends on patient's characteristics and preferences. 3 Penile reconstruction has good outcomes in terms of quality of life and sexual function. 4 5

The radial forearm flap is the gold standard for phalloplasty and penile reconstruction based on its thinness, pliability, reliable vascularity, long pedicle, and potential for resensibilisation. 2 3 4 6 It also provides enough tissue for tubularization and urethroplasty in the same procedure. 3 However, it has an unfavorable donor-site scar location, a tendency to get thinner over time, some color mismatch, and the absence of collateral circulation of the hand is a contraindication. 4 7 8 It has a complication rate of 41% (urethrocutaneous fistulas, urinary strictures). 3 6 9

During the past years, there has been an increased interest in alternative flaps for penile reconstruction, namely, the ALT flap. It was first described as a pedicled flap in 2005 by Ceulemans 10 and later as a free flap by Felici and Felici. 11 It is a reliable alternative with good aesthetic and functional outcomes, and has the advantages of a more favorable donor site, a better color match, the potential for a longer phallus and the possibility of being pedicled avoiding microsurgical procedures. 4 8 It also has the potential for resensibilization if the lateral femoral cutaneous nerve is coapted to the pudendal nerve or dorsal penile nerve. 3 Its limitations include its thickness and vascular anatomy variability, and its higher rates of complications compared with the radial forearm free flap. 4 7 8 12 Multiple revisional surgeries are usually needed for debulking and glansplasty. 8

ALT flap reconstruction can include a urethroplasty: it can be achieved either by using a tube-in-tube technique (like in radial forearm flap), by using a second flap (such as the superficial circumflex iliac artery perforator flap and the radial forearm flap), or by using skin or mucosa grafts. 4 7 8 Using a skin flap for urethroplasty provides better results than using mucosa or skin grafts. 13 14 15

Other flap-based reconstructive techniques include free fibular flap, thoracodorsal artery perforator flap, and pedicled suprapubic abdominal wall flap. 3 New alternatives to flap reconstruction can be provided by tissue engineering (using acellular collagen matrices, 16 autologous cartilage rods, 17 18 and muscle-derived stem cells 19 20 ).

Carbapenemase-producing Enterobacterales is a group of highly drug-resistant gram-negative bacilli causing infections. They have emerged as a significant global public health problem that places patients at risk of potentially untreatable infection, being carbapenemase-producing *Klebsiella pneumoniae* (KPC) the most common and already a reality in plastic surgery departments. 21 KPC infection may be specially problematic if present in chronic wounds and complex fistulizing disease.

In our patient, KPC infection was probably facilitated because of multiple treatments in different hospitals. ALT flap provided vascularized tissue transfer to a very sick, infected, hypoxic, and chronic wound. It also provided continuity with the surrounding cutaneous tissue, leading to better venous and lymphatic drainage and a better cosmetic appearance. 1 22 The fascia lata was transferred and combined to the ALT flap as a vascularized graft, receiving its blood supply via the prefascial and subfascial vascular plexus. 1 Since it has a pliable and stretchable structure with no hair, it was also described to create a tube-shaped neourethra 1 avoiding postoperative complications such as infection, stone formation, fistula, and strictures. In our case since urethral reconstruction was not an option, fascial lata was used for used for Buck's fascia replacement and to prevent infection progression, because of the rich subcutaneous vascular plexus and viability not depending on the quality of the recipient bed, while reducing risks in subsequent secondary surgical procedures. To our best knowledge, composite ALT flap and vascularized fascia lata have not been previously reported for salvage of a near-total penile amputation following urinary fistulization and KPC infection.

Most frequent complications of penile reconstruction include partial or complete flap loss, infection, and delayed wound healing. 8 Complete flap loss is rare. Partial flap loss usually occurs in the distal tip or base of the flap and requires debridement. Infections may occur as soft tissue infections or urinary tract infections. Delayed wound healing occurs most commonly at the ventral base of the penis since it is a place of confluence of multiple suture lines and is usually wet, and are left to heal by secondary intention. 8

Our patient had a favorable evolution. He needed two minor revision procedures: one to debride remaining residual nonviable tissue associated with perineal urethroplasty and other for surgical wound revision. The flap remained viable, with good aesthetic results. Standing voiding function was not restored since the patient had a perineal urethrostomy during the second procedure. No major complications were observed. However, erectile function was not present probably in consequence of extensive soft tissue damage, and penile prosthesis insertion is being considered. Fat defatting/thinning was unnecessary and the patient was satisfied with the flap bulk and donor-site scar.

Transurethral resection of prostate is a standard procedure for refractory lower urinary tract symptoms. Although relatively safe, uncommon complications such as panurethral necrosis, strictures, followed by prostatosymphyseal, and rectourethral fistulas have been reported. 23 24 However, panurethral fistulization is even more rare. The reconstructive surgeon should be aware of this kind of complications, and penile reconstruction in this context should involve a multidisciplinary team, requiring a combined urologic and plastic surgical approach. A psychological evaluation is also important since such defects usually affect patient's psychological well-being and relationships.

Harvesting of the pedicled ALT flap combined with vascularized fascia lata may allow functional and aesthetic reconstruction of complex penile defects, and should be considered as a salvage procedure to avoid penile amputation in chronic infected and intractable wounds.
